# Citrate Transporter Expression and Localization: The *Slc13a5*^Flag^ Mouse Model

**DOI:** 10.3390/ijms26146707

**Published:** 2025-07-12

**Authors:** Jan C.-C. Hu, Tian Liang, Hong Zhang, Yuanyuan Hu, Yasuo Yamakoshi, Ryuji Yamamoto, Chuhua Zhang, Hui Li, Charles E. Smith, James P. Simmer

**Affiliations:** 1 Department of Biologic and Materials Sciences & Prosthodontics, University of Michigan School of Dentistry, 1011 N University Ave., Ann Arbor, MI 48109, USA; zhanghon@umich.edu (H.Z.); chuhuaz@umich.edu (C.Z.); lihuium@umich.edu (H.L.); charles.smith@mcgill.ca (C.E.S.); jsimmer@umich.edu (J.P.S.); 2 Department of Orthodontic and Pediatric Dentistry, University of Michigan School of Dentistry, 1011 N University Ave., Ann Arbor, MI 48109, USA; tianl@umich.edu (T.L.); yyhu@umich.edu (Y.H.); 3 Department of Biochemistry and Molecular Biology, School of Dental Medicine, Tsurumi University, 2-1-3 Tsurumi, Tsurumi-ku, Yokohama 230-8501, Japan; yamakoshi-y@tsurumi-u.ac.jp (Y.Y.); yamamoto-rj@tsurumi-u.ac.jp (R.Y.); 4 Department of Anatomy & Cell Biology, Faculty of Medicine & Health Sciences, McGill University, 3640 University Street, Montreal, QC H3A 0C7, Canada

**Keywords:** enamel mineralization, amelogenesis, protein localization

## Abstract

The sodium–citrate cotransporter (NaCT) plays a crucial role in citrate transport during amelogenesis. Mutations in the *SLC13A5* gene, which encodes the NaCT, cause early infantile epileptic encephalopathy 25 and amelogenesis imperfecta. We analyzed developing pig molars and determined that the citrate concentrations in secretory- and maturation-stage enamel are both 5.3 µmol/g, with about 95% of the citrate being bound to mineral. To better understand how citrate might enter developing enamel, we developed *Slc13a5*^Flag^ reporter mice that express NaCT with a C-terminal Flag-tag (DYKDDDDK) that can be specifically and accurately recognized by commercially available anti-Flag antibodies. The 24-base Flag coding sequence was located immediately upstream of the natural translation termination codon (TAG) and was validated by Sanger sequencing. The general development, physical activities, and reproductive outcomes of this mouse strain were comparable to those of the C57BL/6 mice. No differences were detected between the *Slc13a5*^Flag^ and wild-type mice. Tooth development was extensively characterized using dissection microscopy, bSEM, light microscopy, in situ hybridization, and immunohistochemistry. Tooth formation was not altered in any detectable way by the introduction of the Flag. The *Slc13a5*^Flag^ citrate transporter was observed on all outer membranes of secretory ameloblasts (distal, lateral, and proximal), with the strongest signal on the Tomes process, and was detectable in all but the distal membrane of maturation-stage ameloblasts. The papillary layer also showed positive immunostaining for Flag. The outer membrane of odontoblasts stained stronger than ameloblasts, except for the odontoblastic processes, which did not immunostain. As NaCT is thought to only facilitate citrate entry into the cell, we performed in situ hybridization that showed *Ank* is not expressed by secretory- or maturation-stage ameloblasts, ruling out that ANK can transport citrate into enamel. In conclusion, we developed *Slc13a5*^Flag^ reporter mice that provide specific and sensitive localization of a fully functional NaCT-Flag protein. The localization of the *Slc13a5*^Flag^ citrate transporter throughout the ameloblast membrane suggests that either citrate enters enamel by a paracellular route or NaCT can transport citrate bidirectionally (into or out of ameloblasts) depending upon local conditions.

## 1. Introduction

The human solute carrier family 13 consists of five transmembrane proteins mediating the transportation of two types of substrates, either sulfate or di-/tricarboxylates. All five *SLC13* genes encode sodium-coupled transporters for anions and share approximately 40–50% protein sequence identity [1]. Among these five members, the transporters NaS1 and NaS2 are specialized in carrying substrates such as selenate, sulfate, and thiosulfate. The remaining members of the family, NaDC1, NaDC3, and NaCT, transport di- and tricarboxylates including citrate, succinate, and α-ketoglutarate. Notably, NaCT encoded by *SLC13A5* is a sodium-coupled citrate transporter expressed in liver, brain, and testis which mediates citrate influx from extracellular spaces into the cells to facilitate and maintain cellular metabolic homeostasis.

In mammalian cells, the Na^+^–citrate transporter, NaCT, is generally located on plasma membranes. This transporter was first identified in the human brain. It is also highly expressed in human liver and widely detected in rodent liver, brain, testis, kidney, and muscle [2,3]. It is generally held that NaCT preferentially mediates the influx of citrate into cells from the circulation [3], transports citrate in its trivalent form together with four sodium ions, and functions at an optimal pH of seven [4]. The functionality of NaCT is essential to glucose metabolism and lipogenesis in the liver. Its deletion or inhibition has shown protective effects against high-fat-diet-associated insulin resistance, making the development of NaCT inhibitors of significant therapeutic interest [5,6,7]. Biallelic (recessive) mutations in the human *SLC13A5* gene cause developmental and epileptic encephalopathy 25 with amelogenesis imperfecta (DEE25, MIM #615905). No normal enamel forms on teeth in the absence of NaCT [8]. 

In humans, the normal plasma citrate concentration is within a range of ~100–150 µM [9]. Citrate plays an important role in the mineralization of bones and teeth [10]. About 90% of total citrate in the body resides in the bones. About 1.2 g of citrate is present in 100 g of dried facial bone (% dry wt.). The % dry weight of citrate in dentin and cementum is 0.9 g, and in dental enamel, it is 0.1 g [11]. In bone, citrate is integrally bound to apatite nanocrystals, influencing bone’s structural integrity and resistance to fractures. This phenomenon is highly conserved across fish, birds, and mammals [12,13,14]. While citrate is present in the developing enamel [15], how it gets there and its role are largely unknown. The complete absence of dental enamel when *SLC13A5* is mutated supports that both citrate and its transporter are functionally critical for amelogenesis.

To understand the specific roles of NaCT during amelogenesis, we set out to determine the spatial and temporal expression of this transporter. We hypothesized that defining the expression and localization of NaCT during tooth development would shed light on citrate influx in ameloblasts and its potential role in enamel mineralization. Numerous anti-NaCT antibodies are available from commercial sources; however, their cross-reactivity with different SLC13 family members limits the specificity of these antibodies. To overcome these problems, we designed, generated, and validated an *Slc13a5* Flag-tagged mouse model. Our objective was to generate a stable mouse model that would allow the strong and specific localization of NaCT protein expression wherever it is normally expressed.

## 2. Results

**Figure 1:**
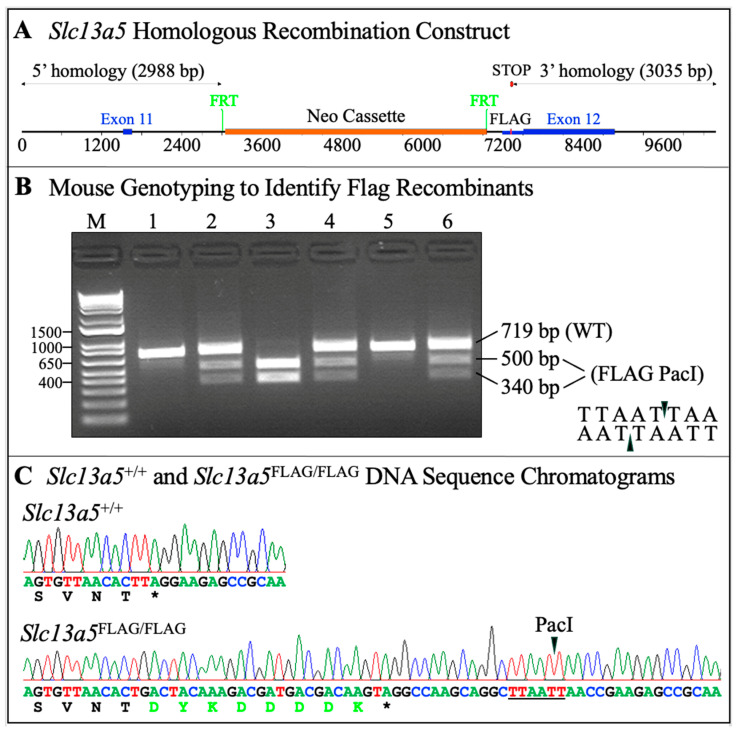
Generation and validation of the *Slc13a5*^Flag^ knock-in mouse model. (**A**) The gene targeting construct used a Neo (neomycin) cassette selection marker flanked by FRT sites inserted into intron 11 of mouse *Slc13a5*. Note: Flp recombinase-mediated recombination occurs between these FRT sites. A Flag-tag (DYKDDDDK) coding sequence (GACTACAAAGACGATGACGACAAG) was introduced immediately upstream of the natural TAG translation termination codon in exon 12 using homologous recombination. (**B**) To identify *Slc13a5*^Flag^ mice, the 5′ ends of the *Slc13a5* and *Slc13a5*^Flag^ genes were amplified from tail biopsies with a primer pair that, following PacI restriction, yielded an uncut 719 bp product from the wild type and an 840 bp knock-in product cut into 500 and 340 bp fragments that were visualized by agarose gel electrophoresis. The gel image demonstrated two wild-type mice (lanes 1 and 5), three heterozygous mice *Slc13a5^+/^*^Flag^ (lanes 2, 4, and 6), and one homozygous *Slc13a5*^Flag/Flag^ in lane 3. (**C**) *Slc13a5*^+/+^ and *Slc13a5*^Flag/Flag^ sequence chromatograms showing 48 nucleotides replaced the TAG stop codon and added code for the 8-amino-acid Flag-tag (shown in fluorescent green) and a downstream PacI restriction site (for ease of genotyping).

Using a homologous recombination strategy, a knock-in *Slc13a5*^Flag^ mouse model was correctly constructed to precisely localize the NaCT-Flag protein in vivo (Figure 1A and Figure S1). Heterozygous mice were bred to generate *Slc13a5*^+/+^, *Slc13a5*^+/Flag^, and *Slc13a5*^Flag/Flag^ offspring (Figure 1B). The targeted sequence modification (*Slc13a5*^Flag^) was correctly introduced and confirmed by Sanger sequencing (Figure 1C). Growth and development, physical activities, and reproductive outcomes were evaluated. The *Slc13a5*^+/+^, *Slc13a5*^+/Flag^, and *Slc13a5*^Flag/Flag^ mice showed no detectable differences.

The body size of mice from this strain at various stages of development was not different from that of wild-type mice. The average litter size was 7.3 pups from nine heterozygous breeding cages which produced 20 litters over a 6-month period. During this period, the C57B/6 litter size was 7.8 from 12 breeding cages which generated 140 pups in 18 litters during the same period. The male-to-female pup ratio was comparable to that of the wild type. The genotype distribution of pups from heterozygous male and female breeding followed the ratio of 50% heterozygous *Slc13a5^+^*^/Flag^, 25% wild-type *Slc13a5^+/+^*, and 25% homozygous for *Slc13a5*^Flag/Flag^.

**Figure 2:**
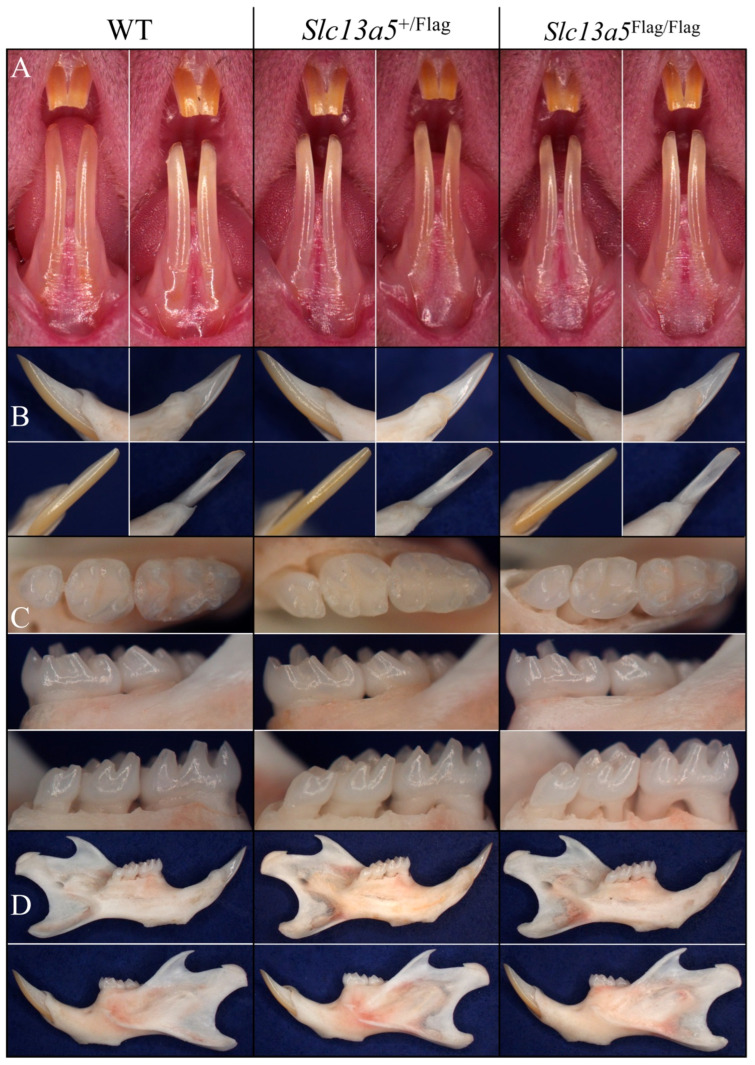
Dissection microscope images of *Slc13a5*^+/+^, *Slc13a5*^+/Flag^, and *Slc13a5*^Flag/Flag^ mouse incisors, molars, and hemimandibles at 7 weeks. (**A**) Labial view of incisors from two mice of each genotype. (**B**) Going clockwise from upper right are mesial, distal, lingual, and labial views of a mandibular incisor. (**C**) Occlusal (top), buccal, and lingual (bottom) views of molars. (**D**) Lingual (top) and lateral (bottom) views of a hemimandible from each genotype. No differences were observed at 7 weeks among the WT (*Slc13a5*^+/+^), heterozygous (*Slc13a5*^+/Flag^), and homozygous (*Slc13a5*^Flag/Flag^) mandibular incisors, molars, or mandible.

*Slc13a5*^Flag^ mice showed completely normal enamel (Figure 2). The size, shape, and color of the incisors and molars among the 7-week-old *Slc13a5*^+/+^, *Slc13a5*^+/Flag^, and *Slc13a5*^Flag/Flag^ mice were indistinguishable. The expression of the *Slc13a5*^Flag^ gene in newborn *Slc13a5*^+/Flag^ mice was assessed in the molars and submandibular salivary glands, while seminal vesicles and testes were dissected from 35-week-old *Slc13a5*^Flag/Flag^ mice. Using primers containing *Slc13a5* and the Flag coding sequence for RT-PCR, the *Slc13a5* transcript was expressed in the molars and testes but not in the salivary glands or seminal vesicles (Figure S3).

This mouse model was successfully used to detect *Slc13a5*^Flag^ expression and localization. Following validation and characterization, this mouse model, B6-Slc13a5 knock-in Flag tag, was preserved at the Mutant Mouse Resource and Research Center (MMRRC), ID: 68041 B6.Cg-*Slc13a5^tm1Jcch^*/Mmnc.

**Figure 3:**
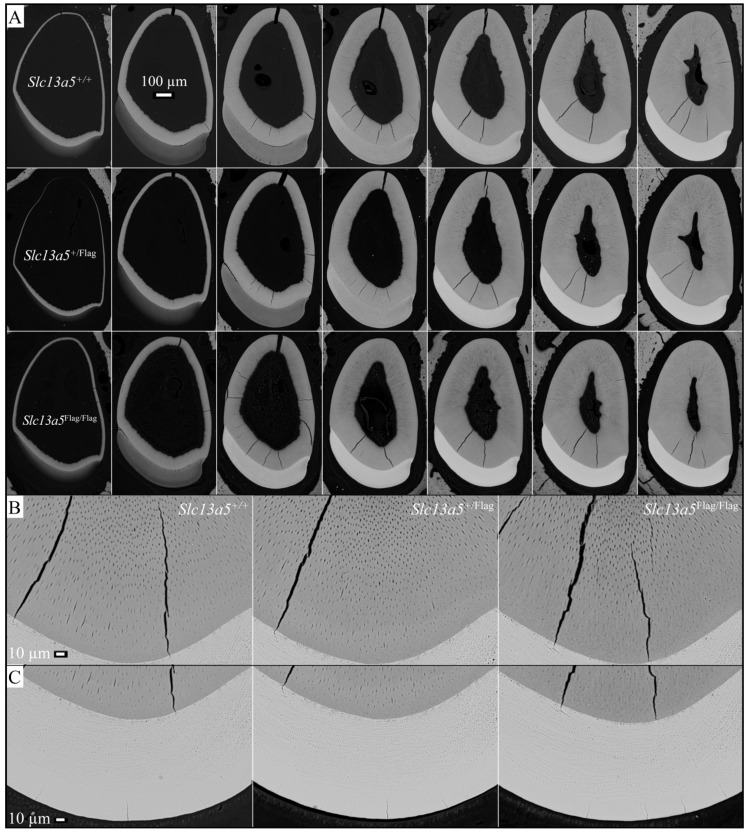
Backscattered scanning electron microscopy (bSEM) of *Slc13a5*^+/+^, *Slc13a5*^+/Flag^, and *Slc13a5*^Flag/Flag^ 7-week mandibular incisors cross-sectioned at 1 mm increments. (**A**) The incisor sections are arranged in a time-forward developmental sequence from left (cervical/earliest development) to right (incisal/more advanced development). The secretory stage of enamel formation (when enamel steadily expands in cross-sectional area) is complete or nearly complete in the second column of cross-sections. By the third column, enamel formation is entirely in the maturation stage where the cross-sectional area of enamel is constant but becomes progressively more mineralized (whiter). Dentin (surrounding the radiolucent dental pulp in the center) continues to increase in cross-sectional area by the further deposition of dentin by odontoblasts lining the pulp. Dentin is less mineralized than enamel and contains characteristic dentinal tubules occupied by odontoblast processes. (**B**) Higher magnification images showing the dentinal tubules nearest the dentinoenamel junction (DEJ). (**C**) Higher magnification images of the highly mineralized enamel. The heterozygous (*Slc13a5*^+/Flag^) and homozygous (*Slc13a5*^Flag/Flag^) mandibular incisors were indistinguishable from the wild type (*Slc13a5*^+/+^).

**Figure 4:**
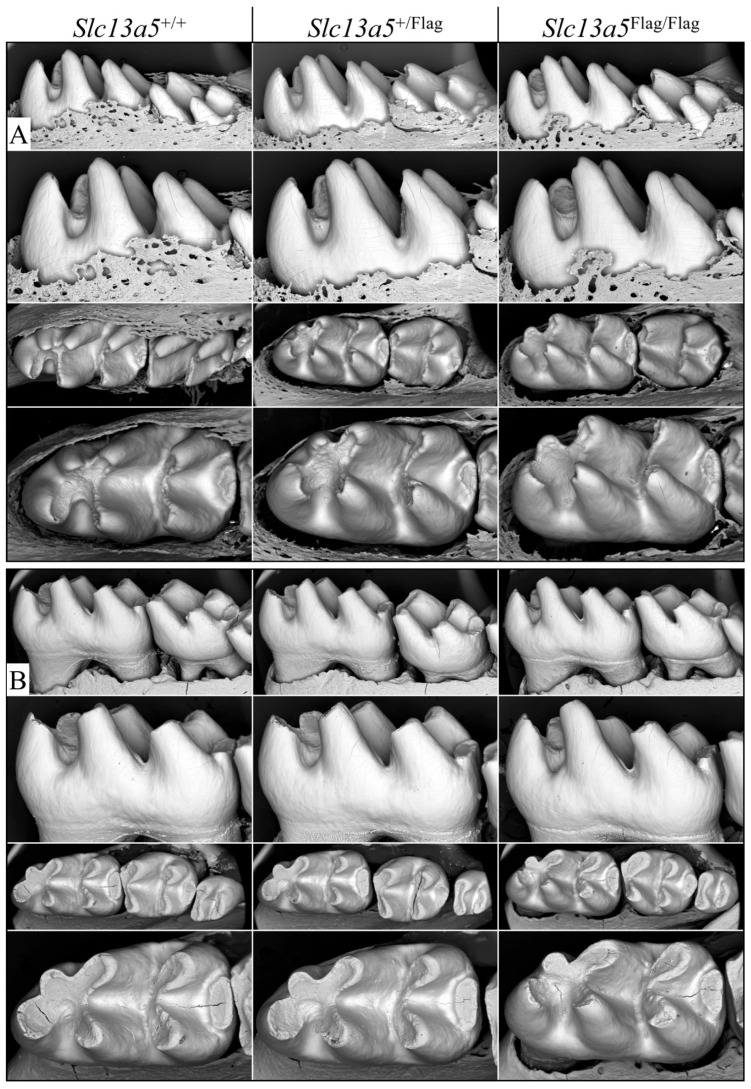
*Slc13a5*^+/+^, *Slc13a5*^+/Flag^, and *Slc13a5*^Flag/Flag^ mandibular mouse molars evaluated by bSEM. (**A**) Day 14 mandibular first molars immediately prior to their eruption through soft tissue into the oral cavity. The crowns are fully formed but have not yet been altered by occlusal forces. (**B**) Molar crowns at 7 weeks that had been functioning for ~5 weeks. The heterozygous (*Slc13a5*^+/Flag^) and homozygous (*Slc13a5*^Flag/Flag^) mouse teeth were indistinguishable from the wild type (*Slc13a5*^+/+^).

Wild-type, heterozygous, and homozygous Flag-tag mouse mandibular incisors were characterized using backscattered scanning electron microscopy (bSEM). All three genotypes were indistinguishable, with no perceivable differences in the enamel and dentin shape, dimensions, or degree of mineralization (Figure 3). Mandibular molars were also characterized by bSEM, at D14 immediately prior to the first molar erupting into occlusion (when its structure could be altered) to assess molar form at the completion of amelogenesis and at 7 weeks when the ability of enamel to withstand weeks in function is apparent (Figure 4). The mandibular molars of the three genotypes (Figure 4A) at D14 were identical in form, indicating that the enamel had achieved its normal thickness and texture. The molars had also undergone normal occlusal wear at 7 weeks (Figure 4B), displaying no structural weaknesses that would have been apparent in the absence of normal NaCT function.

**Figure 5:**
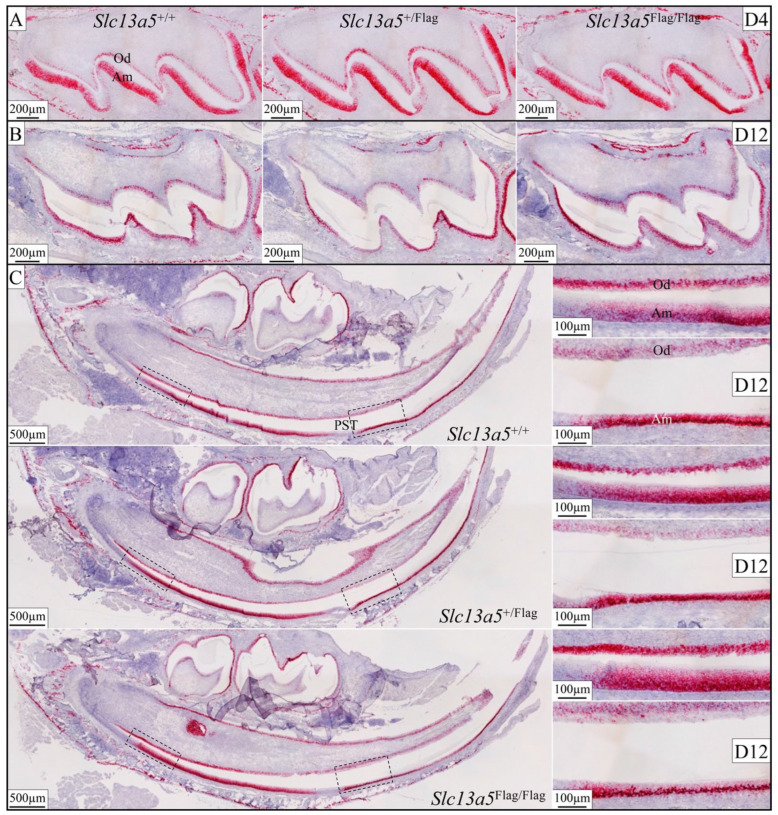
In situ hybridization shows *Slc13a5* mRNA expression is not altered by the insertion of the Flag sequence. (**A**) *Slc13a5*^+/+^, *Slc13a5*^+/Flag^, and *Slc13a5*^Flag/Flag^ Day 4 maxillary first molars, in which all ameloblasts are still in the secretory stage and show comparable patterns of *Slc13a5* expression in both ameloblasts (Ams) and odontoblasts (Ods). (**B**) *Slc13a5*^+/+^, *Slc13a5*^+/Flag^, and *Slc13a5*^Flag/Flag^ Day 12 maxillary first molars, in which all ameloblasts have transitioned into the maturation stage and show the same patterns of *Slc13a5* expression in ameloblasts and odontoblasts. (**C**) Ameloblasts in post-secretory transition (PST) were negative for *Slc13a5* mRNA expression. Mandibular cross-sections are on the left. Dashed rectangles on the left in these sections correspond to odontoblasts above early-secretory-stage ameloblasts in the higher magnification sections (top right). Dashed rectangles on the right correspond to odontoblasts above early-maturation-stage ameloblasts in the higher magnification sections (bottom right). *Slc13a5*^+/+^, *Slc13a5*^+/Flag^, and *Slc13a5*^Flag/Flag^ Day 12 mandibular incisors all showed the same patterns of *Slc13a5* expression.

The expression of *Slc13a5* mRNA in D4 maxillary molars (Figure 5A,B and Figure S2) and in D12 maxillary molars and mandibular incisors (Figure 5B,C) was evaluated using in situ hybridization. There were no detectable differences in intensity or spatial and temporal expression patterns among the *Slc13a5*^+/+^, *Slc13a5*^+/Flag^, and *Slc13a5*^Flag/Flag^ mice (Figure 5 and Figure S3). The positive expression pattern and intensity were consistent in odontoblasts and osteoblasts in the alveolar bone. *Slc13a5* expression in ameloblasts persisted from the onset of the secretory stage to the late maturation stage, with a notable and specific lack of expression during the brief post-secretory transition between the secretory- and maturation-stage ameloblasts (Figure 5). To validate the RT-PCR results, in situ hybridization experiments were conducted on D4 maxillary molars and 2-month-old wild-type mouse testes. Positive signals were consistently detected in the testes, specifically in the spermatocytes (Figure S4).

**Figure 6:**
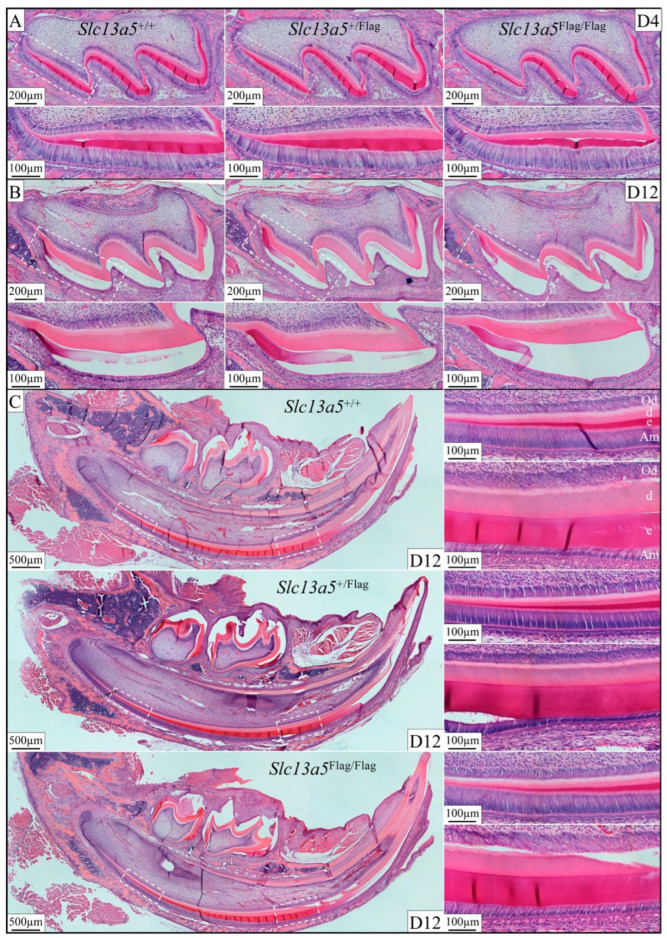
Incisor and molar development was not altered histologically by the insertion of the Flag sequence into *Slc13a5*. (**A**) H&E-stained postnatal Day 4 (D4) maxillary first molars showing overall crown morphology (top) and higher magnification sections from dashed regions (bottom). Note: the morphologies of secretory-stage ameloblasts, odontoblasts, and the dentin and enamel layers are all comparable. (**B**) Comparable images of D12 maxillary first molars during the maturation stage. Overall molar shape root development, dentin, and maturation-stage enamel layers are comparable. Higher magnification panels (bottom) of the boxed areas show that the odontoblasts and maturation-stage ameloblast morphologies are all normal. (**C**) Longitudinal sections of D12 mandibular incisors were similar among the three genotypes (left). Higher magnifications of the odontoblasts (Od), dentin (d), enamel (e), and ameloblasts (Am) showed normal cell morphology and deposition of mineral.

To assess the tissue and cellular integrity, H&E-stained histological sections of D4 and D12 maxillary molars and mandibular incisors were compared among the *Slc13a5*^+/+^, *Slc13a5*^+/Flag^, and *Slc13a5*^Flag/Flag^ mice (Figure 6). Normal development with comparable morphology among samples from the three genotypes was observed. Additionally, the enamel and dentin depositions were no different during both the secretory and the maturation stages among the three genotypes. The closer view of the secretory-stage ameloblast, enamel, dentin matrix, and odontoblast (Figure 6C) shows stage-appropriate development.

**Figure 7:**
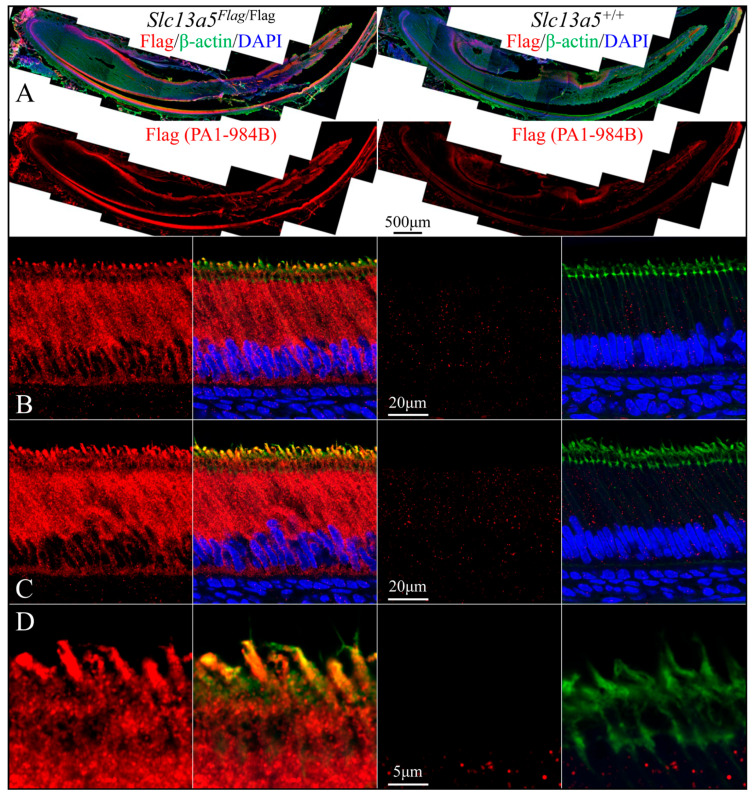
Immunohistochemistry of Day 12 longitudinal sections of *Slc13a5*^Flag/Flag^ (left) and wild-type (right) mandibular incisors triple-stained using PA1-984B antibodies against the Flag-tag on the NaCT (red), β-actin antibodies (green) to stain the ameloblast Tomes processes, and DAPI (blue) to stain nuclei. (**A**) Low-magnification views of mandibular incisors. Top: incisor sections stained for Flag (red), β-actin (green), and nuclei (blue). Bottom: incisor sections showing only Flag stain. Note: ameloblasts were positively stained for NaCT-Flag (left), with a minor background signal observed in the wild type (right, where no Flag is expressed). (**B**) High-magnification images of early-secretory-stage ameloblasts positive for Flag (left) and triple-stained (right). (**C**) Mid-secretory-stage ameloblasts stained heavily for the NaCT (left), and this signal overlapped with β-actin in the Tomes process (right). (**D**) The highest magnification views of the positive signal for NaCT-Flag in the Tomes processes. Note the minimal background of the positive Flag signal in the wild-type sections, which did not express the Flag protein.

**Figure 8:**
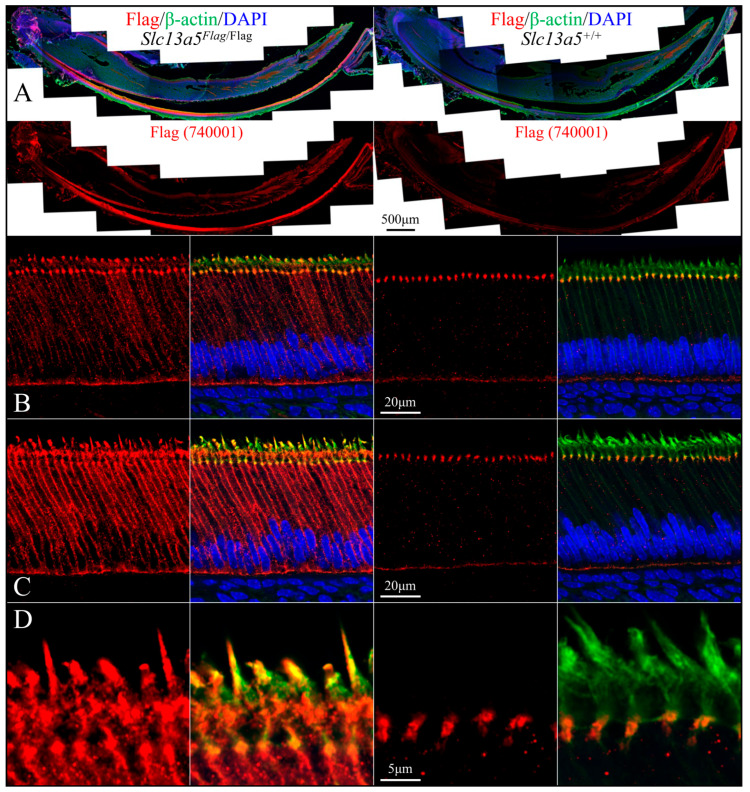
Immunohistochemistry of Day 12 longitudinal sections of *Slc13a5*^Flag/Flag^ (left) and wild-type (right) mandibular incisors triple-stained using 740001 antibodies against the Flag-tag on the NaCT (red), β-actin antibodies (green) to stain the ameloblast Tomes processes, and DAPI (blue) to stain nuclei. (**A**) Low-magnification views of entire incisors. Top: incisor sections stained for Flag (red), β-actin (green), and nuclei (blue). Bottom: incisor sections showing only Flag signal. Note: ameloblasts were positive for NaCT-Flag (left), with some background signal in the wild type (right) associated with intercellular junctions. (**B**) High-magnification images of early-secretory-stage ameloblasts immunostained for Flag (left) and triple-stained (right). (**C**) Mid-secretory-stage ameloblasts stained heavily for the NaCT (left), and this signal overlapped with β-actin in the Tomes process (right). (**D**) The highest magnification views of the positive signal for NaCT-Flag in the Tomes processes. Note the background of the positive Flag signal in wild-type sections (right) was higher than what was observed using the PA1-984B antibody in Figure 7 and Figure 9.

**Figure 9:**
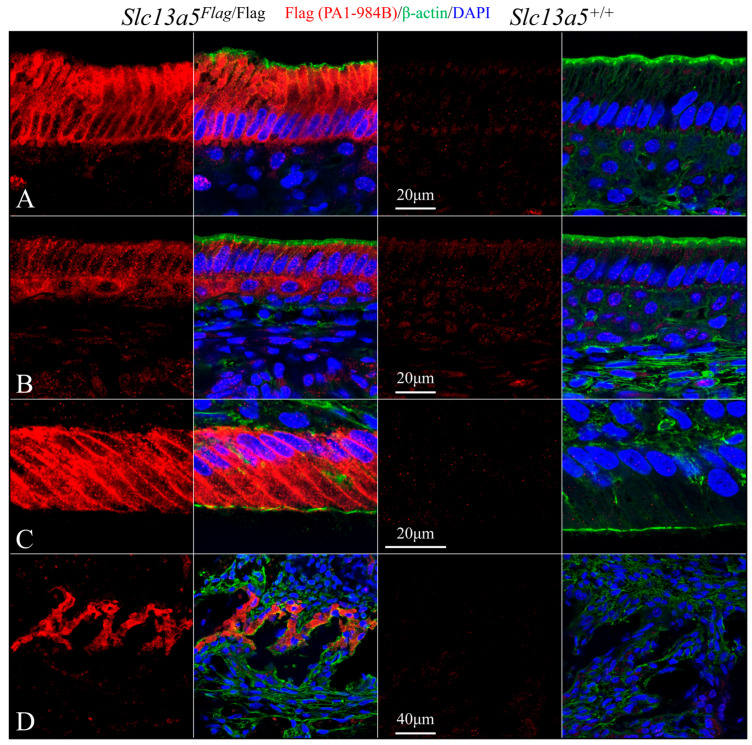
Immunohistochemistry of Day 12 longitudinal sections of *Slc13a5*^Flag/Flag^ (left) and wild-type (right) mandibular incisors triple-stained using PA1-984B antibodies to stain NaCT-Flag (red), β-actin antibodies (green), and DAPI (blue) to stain nuclei. (**A**) Early-maturation-stage ameloblasts (lacking Tomes processes) immunostained for Flag only (left) and triple-stained (right). NaCT-Flag localizes throughout the cell membrane of the maturation-stage ameloblasts. Note the minimal background of the positive Flag signal in wild-type sections (right). (**B**) Late-maturation-stage ameloblasts and adjacent cells of the papillary layer showed positive immunostaining for Flag. (**C**) Odontoblasts were strongly stained by the NaCT-Flag antibody compared to ameloblasts. The exposure was reduced when acquiring images, which also reduced the background staining. (**D**) Osteoblasts were positively stained by the NaCT-Flag antibody. The (PA1-984B) Flag antibody proved to be both sensitive and specific in detecting Flag among the ameloblasts, odontoblasts, and osteoblasts in the mandible.

**Figure 10:**
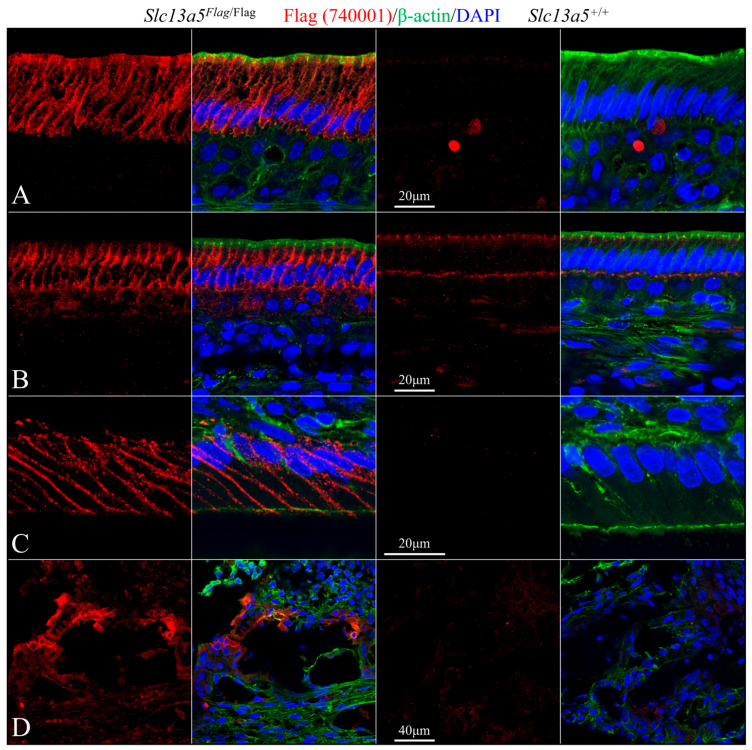
Immunohistochemistry of Day 12 longitudinal sections of *Slc13a5*^Flag/Flag^ (left) and wild-type (right) mandibular incisors triple-stained using 740001 antibodies against the Flag-tag on the NaCT (red), β-actin antibodies (green) to stain the maturation-stage ameloblasts’ distal surfaces (green), and DAPI (blue) to stain nuclei. (**A**) Early-maturation-stage ameloblasts (lacking Tomes processes) immunostained for Flag only (left) and triple-stained (right). NaCT-Flag localized along the cell membranes of the maturation-stage ameloblasts. Note the minimal background of the positive Flag signal in wild-type sections (right). (**B**) Late-maturation-stage ameloblasts and adjacent cells of the papillary layer showed positive immunostaining for Flag. The background staining of the cell junctions was evident at this stage. (**C**) Odontoblasts were strongly stained by the NaCT-Flag antibody compared to the ameloblasts. The exposure was reduced when acquiring the images, which also reduced the background staining. (**D**) Osteoblasts were positively stained by the NaCT-Flag antibody.

These extensive analyses provided conclusive evidence that introducing the Flag sequence immediately before the translation stop codon did not alter either the temporal or spatial expression of *Slc13a5*, the stability of its mRNA transcript, or the function of the NaCT-Flag protein during odontogenesis. We then turned to using Flag antibodies to localize the NaCT-Flag protein in vivo in the continuously growing mandibular incisor, which includes all stages of dentin and enamel formation/mineralization (Figure 7, Figure 8, Figure 9 and Figure 10).

We used rabbit anti-DYKDDDDK recombinant protein polyclonal antibodies PA1-984B (Figure 7 and Figure 9) and 740001 (Figure 8 and Figure 10) from ThermoFisher Scientific to define the subcellular localization of the NaCT-Flag in ameloblasts and odontoblasts during tooth development (Figure 7, Figure 8, Figure 9 and Figure 10). To clearly distinguish the true Flag signal (NaCT protein) from background staining, each figure compares mandibular incisor sections from *Slc13a5*^Flag/Flag^ (left) and *Slc13a5*^+/+^ (right) mice. By convention, for mandibular mouse incisors, the ameloblasts are oriented with their distal ends that interact with the forming enamel matrix at the top and their proximal ends that obtain nutrients from the blood supply at the bottom. The pointed structures (Tomes processes) protruding into developing enamel that elongate the enamel mineral ribbons are highlighted by immunostaining for β-actin (green, columns 2 and 4), while the nuclei polarized to the proximal end of each ameloblast are stained with DAPI (blue, columns 2 and 4) (Figure 7 and Figure 8).

Secretory-stage ameloblasts show the strongest NaCT-Flag signal in their Tomes process that elongates enamel mineral ribbons to expand the enamel layer as the ameloblasts slowly retreat proximally (Figure 7 and Figure 8). This localization indicates that citrate is transported across the ameloblast distal membrane, lining the space where enamel forms. There is also a strong, punctate signal throughout the length of the cell (Figure 7) that highlights the long lateral and shorter proximal membranes (Figure 8), implying citrate is transported across the ameloblast lateral membrane associated with intercellular spaces and proximal membrane nearest the blood supply. The Flag signal (and therefore the abundance of NaCT protein) is lower in early-secretory-stage ameloblasts (Figure 7B and Figure 8B) relative to mid-secretory-stage ameloblasts (Figure 7C,D and Figure 8B,C).

The Tomes process is fully retracted during the completion of the secretory stage when ameloblasts terminate the elongation of enamel mineral ribbons and the developing enamel layer achieves its final dimensions but not its final hardness. Maturation-stage ameloblasts modulate between ruffle- and smooth-ended morphologies as the mineral ribbons grow in width and thickness (rather than in length) to harden the enamel [16]. In early-maturation-stage ameloblasts, the NaCT-Flag signal outlined the cells, being evident in the distal (top), lateral, and proximal (bottom) membranes (Figure 9A and Figure 10A). In mid-maturation ameloblasts, the NaCT-Flag signal concentrated in the lateral plasma membrane with minimal signal in the distal (top) and proximal (bottom) membranes. The NaCT-Flag signal was also observed in the papillary layer cells primarily abutting against the modulating ameloblasts (Figure 9B and Figure 10B). At the same time, NaCT-Flag localization in odontoblasts was concentrated in the lateral plasma membranes, with no detectable signal observed in odontoblastic processes that extend into predentin and dentin (Figure 9C and Figure 10C). The NaCT-Flag signal in odontoblasts was notably stronger than in ameloblasts. In the alveolar bone surrounding the developing tooth, a positive NaCT-Flag signal was detected in osteoblasts (Figure 9D and Figure 10D).

The detection of the Flag-tag using IHC was conducted in testis tissues from two-month-old wild-type mice (Figure S4). The positive staining pattern observed was consistent with the expression pattern demonstrated by in situ hybridization (Figure S4A–G).

The rabbit anti-DYKDDDDK recombinant protein polyclonal antibodies PA1-984B and 740001 both provided strong and specific staining patterns in ameloblasts, odontoblasts, and osteoblasts (Figure 7, Figure 8, Figure 9 and Figure 10) in *Slc13a5*^Flag/Flag^ mice. In *Slc13a5*^+/+^ mice, the 740001 antibody cross-reacted with components in the ameloblast distal terminal web and proximal membrane (Figure 8B–D and Figure 10B). This minor cross-reactivity did not interfere with the localization of the *Slc13a5*^Flag^ protein, as the background could easily be subtracted by referencing the negative control images.

**Figure 11:**
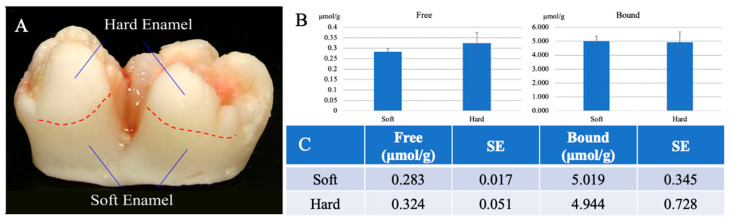
Citrate concentration in porcine hard and soft enamel. Secretory-stage enamel of the developing teeth is relatively soft, whereas maturation-stage enamel is harder. (**A**) Photograph of a 6-month-old porcine mandibular first molar showing the soft and hard enamel. Using six molars from the same developmental stage, the free and bound citrate concentrations were determined. (**B**) Citrate measurements of soft and hard enamel, either bound or free, were comparable. (**C**) The free citrate concentration in soft (secretory-stage) enamel was 0.283 µmol/g, and in hard (maturation-stage) enamel, it was 0.324 µmol/g. The bound citrate concentration was determined to be 5.019 µmol/g and 4.944 µmol/g in the secretory and maturation stage, respectively. There were no statistical differences in the total citrate concentrations between soft and hard porcine enamel. About 94–95% of the citrate is bound to mineral in both stages.

To help make sense of the *Slc13a5*^Flag^ localization, we determined the citrate concentrations in the soft and hard enamel layers of developing 6-month-old porcine mandibular first molars (Figure 11). The PBS-soluble or free citrate concentration of the 6-month-old porcine mandibular first molars’ soft (secretory-stage) and hard (maturation-stage) enamel was determined. Six molars from the same developmental stage were used to assess both the free and bound citrate concentrations. The free citrate concentration was determined to be 0.283 ± 0.017 µmol/g and 0.324 ± 0.051 µmol/g in the secretory and maturation stage, respectively. The bound citrate concentration in secretory-stage enamel was 5.019 ± 0.345 µmol/g, and in maturation-stage enamel, it was similar at 4.944 ± 0.728 µmol/g (Figure 11B,C). There was no statistical difference found in the citrate contents between the soft (secretory-stage) and hard (maturation-stage) enamel using one-way ANOVA (*p* = 0.1174).

Adding the bound and unbound citrate concentrations, the total citrate concentrations in secretory- and maturation-stage enamel are both 5.3 µmol/g. The percentage of citrate molecules that are unbound was also very similar: 5.3% in the secretory stage and 6.2% in the maturation stage.

**Figure 12:**
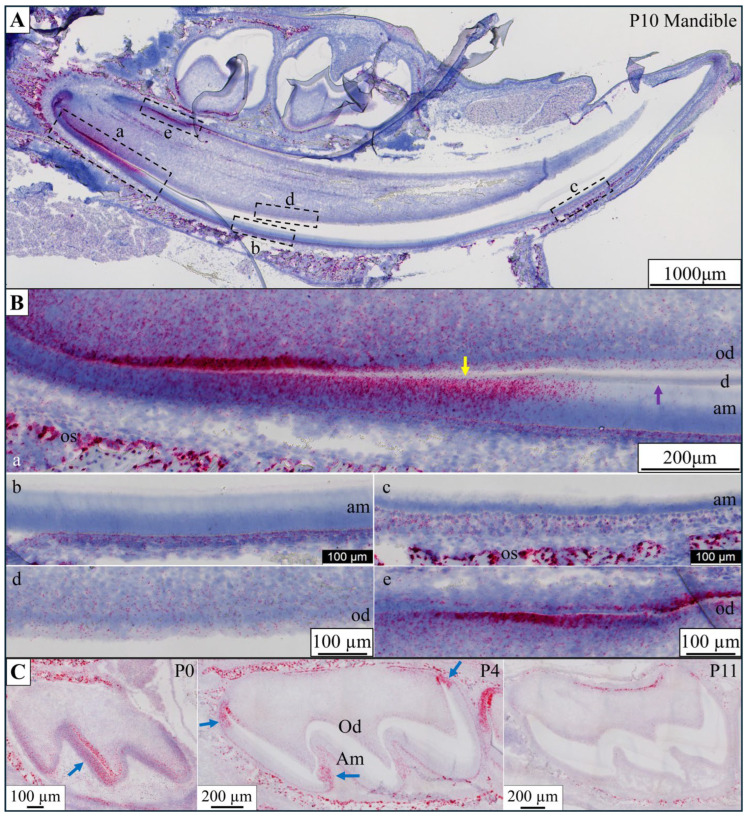
In situ hybridization staining of *Ank* mRNA in D10 mandible and Day 0, 4, and 11 maxillary first molars. (**A**) D10 mandible longitudinal section showing continuously growing incisor containing all stages of ameloblast and odontoblast development. Dashed boxes mark locations of higher magnification images in panel (**B**). (**B**) (**a**) Apical end of D10 mandibular incisor. The positions of the onset of dentin (yellow) and enamel (violet) mineralization are indicated by arrows. Odontoblasts (od) and pre-ameloblasts express *Ank* prior to the onset of dentin and enamel mineralization. Alveolar bone osteoblasts (os) show strong *Ank* expression. (**b**) Secretory-stage ameloblasts (am) and (**c**) maturation-stage ameloblasts are negative for *Ank* transcripts. *Ank* mRNA is strongly detected in alveolar bone osteoblasts. (**d**) Trace expression of *Ank* in odontoblasts beneath secretory-stage ameloblasts. (**e**) Strong *Ank* expression odontoblasts prior to the onset of dentin biomineralization, potentially related to matrix vesicle formation. (**C**) *Ank* in situ hybridization in developing maxillary molars. *Ank* is only transiently expressed by pre-ameloblasts and pre-odontoblasts (blue arrows) in the Day 0 first molar before the onset of mineralization, in the Day 4 molar at the junction of the inner and outer enamel epithelium apically (blue arrows), and in the enamel-free zone at the cusp tip (blue arrow). The Day 11 molar shows no *Ank* signal associated with maturation-stage ameloblasts (Am) and a trace signal in odontoblasts (Od) but a positive signal in osteoblasts in the root bifurcation area.

ANK is a membrane protein capable of transporting citrate into the extracellular matrix [17] and has been proposed to function in ameloblasts for this purpose [18]. We performed in situ hybridization to detect *Ank* mRNA in Day 14 mandibular incisors and maxillary first molars from postnatal Days 0, 4, and 11 mice (Figure 12). In the continuously growing mandibular incisors, which display ameloblasts at all stages of development, no *Ank* mRNA was detected in ameloblasts. Mandibular incisor odontoblasts only transiently expressed *Ank* mRNA immediately prior to the onset of mineralization when matrix vesicles containing ANK initiate dentin mineralization [19]. In developing maxillary molars, *Ank* mRNA was observed in pre-odontoblasts and pre-ameloblasts at Day 0 long before the onset of mineralization and at Day 4 at the junction of the inner and outer enamel epithelium and in the enamel-free zone. A positive signal for *Ank* mRNA was never observed in ameloblasts or odontoblasts during dentin or enamel mineralization.

## 3. Discussion

In the 1950s and 1960s, it was determined that citrate comprises one sixth of the small amount of residual organic material that is present in mature enamel [20,21]. Here, we determined the concentration of citrate in the secretory- and maturation-stage forming enamel of wild-type pigs (Figure 11). The citrate concentration in porcine developing enamel was 5.3–6.2 µmol/g. *SLC13A5* encodes a citrate transporter—the only non-mitochondrial citrate transporter known to be expressed by ameloblasts. Humans lacking a functional NaCT protein show a complete absence of dental enamel [8,22], as do *Slc13a5* KO mice [2,23]. We hypothesize that citrate is essential for dental enamel formation, but an excess of citrate in the developing enamel matrix likely inhibits enamel mineral formation.

Ameloblasts are the only cells in contact with the forming enamel. From their onset on the surface of a recently coalesced, continuous layer of dentin, enamel mineral ribbons elongate in very close proximity to the secretory surface of the ameloblast distal membrane. During the secretory stage of amelogenesis, incoming nutrients from capillaries must pass through the outer enamel epithelium, stellate reticulum, and stratum intermedium and be absorbed by the ameloblast proximal membrane or pass through proximal and distal tight junctions that completely encircle each ameloblast. The tight junctions act as a selective barrier between epithelial cells to regulate the diffusion of materials via the paracellular pathway [24]. Tight junction proteins in the distal (enamel side) and proximal (stratum intermedium side) terminal bar apparatuses are closely associated with intracellular web filaments in secretory-stage ameloblasts [25]. Terminal bar apparatuses participate in the control of the transcellular and extracellular movement of ions and metabolites in the ameloblast layer [26]. The importance of these tight junctions is confirmed by the finding that enamel malformations result from genetic defects in at least five claudin genes: *CLDN1* [27,28], *CLDN3* [29,30], *CLDN10* [31], *CLDN16* [32], and *CLDN19* [33]. However, the terminal bar apparatuses are not completely restrictive but control the transcellular and extracellular movement of ions and metabolites into the ameloblast layer, and the situation concerning the inter- or transcellular transport of citrate specifically has not been characterized.

Our goal in this study was to accurately localize the NaCT protein during the normal process of amelogenesis. Not finding suitable (sensitive and specific) commercial antibodies, *Slc13a5*^Flag^ mice were successfully generated, the proper incorporation of the target sequence was validated, and the enamel layer of these NaCT-Flag-modified mice was thoroughly characterized to determine if the NaCT had been functionally altered by the modifications. The physical assessment of general activities and histological surveys of the organ morphology and tissue/cell integrity of this mouse strain supported that the presence of the Flag-tag did not adversely impact general development, physical activities, reproduction, or tooth development. The expression of the *Slc13a5*^Flag^ transcript can be detected in the developing molars and adult testes but not in the adult seminal vesicles or submandibular salivary glands. The developing dentition was characterized thoroughly at the organ and cellular levels. No structural defects of molars or incisors were observed in *Slc13a5*^Flag^ mice. As *Slc13a5* knock-out mice make no normal enamel and *Slc13a5*^Flag^ mice make enamel indistinguishable from wild-type mice, we conclude that the modification did not disturb NaCT function. The in situ hybridization of wild-type and *Slc13a5*^Flag^ mice showed identical patterns of *Slc13a5* mRNA expression. These results strongly support the conclusion that the cellular localization of the NaCT-Flag protein is the same as that of the wild-type NaCT.

After testing several anti-DYKDDDDK antibodies, we selected rabbit polyclonal antibodies 740001 and PA1-984B from ThermoFisher Scientific for all IHC experiments. The IHC detection of NaCT-Flag in the developing tooth organ demonstrated a positive expression of *Slc13a5*^Flag^ in secretory- and maturation-stage ameloblasts, odontoblasts, and alveolar bone osteoblasts. Of particular interest was the observation that the NaCT-Flag protein is present throughout the entire secretory ameloblast membrane, is most abundant on the Tomes process where enamel ribbons elongate and is increased by the mid-secretory stage. A recent model for the trans-ameloblast transport of citrate proposed that the NaCT is restricted to the ameloblast proximal membrane where it imports citrate, while ANK exports citrate into the developing enamel [21]. This model is not supported by the results of this study, as *Ank* is not expressed by differentiated ameloblasts (Figure 12B). *Ank* is only expressed by early odontoblasts when it is thought to participate in the matrix vesicle-mediated onset of mineralization [19].

The first gene implicated in the etiology of hereditary enamel defects (HEDs) was *AMELX* in 1991 [34]. Since then, genetic malformations affecting enamel have been associated with defects in 115 genes [35]. The success rate for identifying HED-causing genes/mutations using whole-exome sequencing is now over 70%, which limits the search to exons and intron junctions and suggests that there are very few unknown essential genes yet to be discovered. NaCT is the only known citrate transporter associated with hereditary enamel defects. The strong presence of NaCT on the Tomes process and lateral membranes strongly suggest that citrate is transported by the paracellular route into the developing enamel. Alternatively, as we do not know of any other citrate transporter expressed on the outer membrane of ameloblasts [36,37,38], it seems appropriate to consider if the NaCT is capable of transporting citrate *out* of the cell, potentially driven by the higher citrate concentration inside the cell relative to the enamel extracellular matrix.

The five vertebrate SLC13 transporters are electrogenic and produce inward currents in the presence of sodium and substrate [1]. They transport an extracellular ion with multiple negative charges along with one more sodium ion (Na^+^) than the number of negative charges on the substrate, thus generating a membrane potential. NaCT analyses at pH 7.5 gave a value of 3.3 ± 0.3 Na^+^ ions transported per citrate ion [3]. Importantly, the uptake of citrate was inhibited significantly (~43%) when the membrane was depolarized, indicating that citrate uptake is influenced by the membrane potential. This suggested that “at least” four Na^+^ ions are cotransported with one citrate in the transport process, despite this number being outside the margin of error determined experimentally [3]. The cotransport of four Na^+^ ions would enable the transport of citrate against a concentration gradient but would impede the reverse reaction (export of citrate and four Na^+^). It also seems noteworthy that over half of the citrate uptake continued even in the absence of membrane polarization. Is it possible that not all transport cycles transport four sodium ions? The *Xenopus* homologue of SLC13A5 (named *Indy*) is non-electrogenic and can import and export citrate [39].

In this study, the citrate transporter encoded by *Slc13a5* was localized to the cell membrane of ameloblasts, odontoblasts, and osteoblasts, demonstrating a tissue expression pattern consistent with data from the NCBI Gene database. We used adult mouse testes as a positive control and tested salivary glands and seminal vesicles as negative controls for the in situ and IHC experiments. It is curious that despite the high concentration of citrate in seminal fluid [40], the seminal vesicle was negative for *Slc13a5*^Flag^ expression. RT-PCR experiments were conducted with a low number of amplification cycles, and the results were consistently negative for seminal vesicle samples (Figures S2 and S3). In this experiment, *Gapdh* amplification demonstrated that the quality and quantity of the transcripts used for amplifications were comparable among samples.

NaCT functions in cells to mediate citrate intake, which is essential for the metabolic activities of the secretory- and maturation-stage ameloblasts, as reflected by the expression pattern of NaCT-Flag. Based on the results from the citrate assay of developing porcine enamel, free and bound citrate were detected in both the soft (secretory-stage) and hard (maturation-stage) enamel in concentrations of ~5 µmol/g or ~5 mM, which is on the lower end of the range of citrate concentrations in bone and teeth [11]. It is unclear if the tooth samples used to determine the citrate concentration in the 2018 study were the mineralized component alone or included pulp tissues. The citrate content in the developing enamel alone is ~40-fold higher than the citrate concentration of 0.12 mM in blood plasma [9,11,15]. Furthermore, the 2018 study also reported the relative citrate concentrations in muscle (0.04%), cartilage (0.10%), facial bone (1.2%), enamel (0.1%), dentin (0.9%), and cementum (0.9%) as a percentage of dry weight. From the porcine enamel study (Figure 11), a total citrate concentration of 5 µmol/g equals roughly 0.096% by weight, which is consistent with the published data [11].

The high citrate content of the developing porcine enamel is an interesting finding. How did citrate cumulate in the developing enamel matrix? If citrate can pass freely through the proximal and distal terminal bar apparatuses and concentrate in developing enamel, the NaCT on the ameloblast lateral membranes and Tomes process could be pumping excess citrate into ameloblasts to control the citrate concentration in the enamel matrix while contributing to energy production and lipogenesis within the cell. If the terminal bar apparatuses limit the passage of citrate so that citrate must enter the cell and be transported into the developing enamel matrix, then the NaCT must be capable of transporting citrate into and out of ameloblasts, or another citrate transporter must be discovered. To better understand citrate movement and accumulation during amelogenesis, further experiments will be necessary.

While this *Slc13a5*^Flag^ mouse model was primarily developed and characterized to study the localization and movement of citrate during amelogenesis, it also provides a useful resource for researchers interested in examining *Slc13a5*^Flag^ expression in other body systems. Moreover, this model can serve as a valuable platform for therapeutic research, allowing the assessment of the effectiveness of NaCT perturbation or inhibition across various organ systems.

## 4. Materials and Methods

### 4.1. Animal Model Generation and Characterization

This study adhered to the ARRIVE guidelines. The experimental protocol involving the use of laboratory animals was reviewed and approved by the University of Michigan Institutional Animal Care and Use Committees (protocol #PRO00011016). All mice were maintained in a pathogen-free environment with a regulated light cycle and standard rodent chow. When single housing was necessary, mice were provided with enrichment materials. The sample size of each experiment was determined based on prior studies and the relevant literature. For each experiment, at least two males and two females from each genotype were included. Experiments were repeated multiple times to assess reproducibility.

The mouse solute carrier family 13 member 5 [Mus musculus] (sodium-dependent citrate transporter, MGI: 3037150, NM_001004148.4; GI: 281306805; NC_000077.7) gene-modified model was generated in the C57BL/6 background. “Flag” is a highly antigenic eight-amino-acid peptide; its coding sequence can be introduced into a selected gene for the sensitive detection of the target protein expression [41]. Using the homologous recombination approach, the Flag-tag (DYKDDDDK) coding sequence (GACTACAAAGACGATGACGACAAG) was introduced upstream of the natural translation termination codon in *Slc13a5* exon 12 (Figure 1A, Ozgene, Indianapolis, IN, USA). Chimeric mice were produced and bred with C57BL/6J mice to confirm germline transmission. The *Slc13a5*^Flag^ modification was validated by Sanger sequencing. The genotyping primer sets Slc13a5-FLAG-F406, 5′-ACAAAATGGGTGGCAGAAAG-3′, and Slc13a5-FLAG-MU427, 5′-CCAGCATGGA GCCAGTAGTC-3′, were used to generate an amplicon of 719 bp from the wild-type and 840 bp from the mutant samples. The specific reaction conditions included denaturation @ 94 °C for 2 min; then 30 cycles of 94 °C for 30 s (template denaturation), 58 °C for 30 s (primer annealing), and 72 °C for 50 s (primer extension); followed by 72 °C for 2 min; and then hold at 4 °C. Then, the PacI enzyme (New England Biolabs, Ipswich, MA, USA) was used to digest the PCR products. The reaction mix contained Pacl enzyme 0.6 µL, 10× NEBuffer 2.5 µL, PCR product 10 µL, and ddH_2_O 11.9 µL. The reaction mix was incubated at 37 °C for 40 min using a GeneAmp PCR System 9700 Thermocycler (Foster City, CA, USA). The WT amplicon was not digestible by PacI, thus remained as a 719 bp band, while the MU amplicon was digested into 340 bp and 500 bp bands. Thus, the WT and the MU amplicons could be distinguished on a 2% agarose gel (Figure 1B). *Slc13a5*^+/+^ and *Slc13a5*^Flag^ sequence chromatograms showing 48 nucleotides replaced the TAG stop codon, which included nucleotides encoding Flag-tag (shown in fluorescent green) and a downstream PacI restriction site (Figure 1C).

### 4.2. Slc13a5 Transcript Detection

Multiple developing molars were dissected from newborn *Slc13a5*^Flag/Flag^ and D5 *Slc13a5*^+/+^ mice following an appropriate euthanasia protocol. The submandibular salivary glands, seminal vesicles, and testes were dissected from 32-week-old *Slc13a5*^Flag/Flag^ and 7-week-old *Slc13a5*^+/+^ male mice. To detect *Slc13a5* transcripts, two primer sets were used: *Slc13a5+Flag*-F, 5′-GCCAGACTGAGGAAGAAAGG-3′, with *Slc13a5+Flag*-R, 5′-CTTGGCCTACTTGTCGTCATC-3′, which covered exon 8 of transcript variant 1 (TV1) to *Flag* (596 bp amplicon), and set 2: *Gapdh* F, 5′-AGGCCGGTGCTGAGTATGTC-3′, and *Gapdh* R, 5′- TGCCTGCTTCACCACCTTCT-3′ (530 bp amplicon). The PCR reaction included the following steps: denaturation @ 94 °C for 2 min; then 25 cycles of 94 °C for 30 s (template denaturation), 58 °C for 30 s (primer annealing), and 72 °C for 50 s (primer extension); followed by 72 °C for 1 min; and then hold at 4 °C. The amplification products were evaluated on a 2% agarose gel and validated by Sanger sequencing.

### 4.3. Morphological Assessment

To evaluate and compare the phenotype of the developing teeth, multiple 7-week-old *Slc13a5*^+/+^, *Slc13a5*^+/Flag^, and *Slc13a5*^Flag/Flag^ mice were anesthetized and perfused with 4% paraformaldehyde. The hemimandibles were dissected, soft tissues were removed, and teeth and bones were cleaned with 1% sodium hypochlorite, rinsed, air-dried, and displayed under a Nikon SMZ1000 dissection microscope, and images of at least three samples were captured by using a Nikon DXM1200 digital camera (Nikon, Melville, NY, USA).

### 4.4. Backscattered Scanning Electron Microscopy (bSEM)

Seven-week-old hemimandibles from *Slc13a5*^+/+^, *Slc13a5*^+/Flag^, and *Slc13a5*^Flag/Flag^ mice were dehydrated using an acetone series, embedded in epoxy, cross-sectioned at 1 mm increments along the anterior–posterior axis, and characterized by bSEM at each increment. At least three samples from each genotype were analyzed. Furthermore, two- and seven-week-old mice were anesthetized and perfused with 4% PFA. All hemimandibles prepared for surface scanning were carefully dissected free of soft tissue. We used an Amray EF 1910 Scanning Electron Microscope operating at an accelerating voltage of 5 kV to image the samples (MC^2^, North Campus, University of Michigan, Ann Arbor, MI, USA).

### 4.5. Histology, In Situ Hybridization, and Immunohistochemistry

Positive and negative controls were included in all experiments, and experiments were repeated using multiple samples to establish reproducibility. Hemimandibles of 0-, 4-, 11-, and 12-day-old *Slc13a5*^+/+^, *Slc13a5*^+/Flag^, and *Slc13a5*^Flag/Flag^ mice were dissected and fixed in 4% PFA in PBS at 4 °C overnight. The samples were then decalcified at 4 °C in 16.52% diethyl pyrocarbonate-treated disodium ethylenediaminetetra-acetic acid (EDTA, pH 7.4) with agitation for 2 days (D0 and D4 samples) or 5 days (D11 and D12 samples). Samples were then dehydrated through an ethanol series, cleared in xylene, embedded in paraffin, and sectioned at 5 μm thickness using a HistoCore AUTOCUT R microtome (Leica, Deer Park, IL, USA). Sections were loaded onto Fisher Tissue Path Superfrost Plus Gold Microscope Slides (ThermoFisher Scientific, Waltham, MA, USA) for processing.

Routine H&E staining was performed to assess tissue integrity. An RNAscope^®^ ISH probe (Cat#533391, Advanced Cell Diagnostics, Newark, CA, USA) targeting mouse *Slc13a5* mRNA NM_001004148.4 region 41–1023 bp was used to assess the expression of *Slc13a5*. Riboprobes targeting mouse *Ank* mRNA NM_020332.4 region 285–1329 bp (Cat#441181, Advanced Cell Diagnostics) were used to determine the expression of *Ank*. These riboprobes were designed to amplify target-specific signals with reduced background noise from non-specific hybridization [42]. For the negative control, Probe-dapB (Cat#310043, Advanced Cell Diagnostics) was used. The hybridization procedure was conducted following the manufacturer’s recommendations. For image acquisition, we used a Nikon Eclipse TE300 microscope equipped with a Nikon DXM1200 digital camera. For high-resolution imaging, we used the Leica STELLARIS 8 FALCON Confocal Microscopy System at the University of Michigan, Kellogg Eye Center.

Tissue sections underwent an optimized immunohistochemistry protocol [43]. Rabbit-produced affinity-purified anti-Flag antibodies (Sigma-Aldrich F7425, Burlington, MA, USA; ThermoFisher Scientific 740001 and PA1-984B, Waltham, MA, USA) were used to target the 8-amino-acid Flag epitope at the C-terminus of the NaCT. After the incubation with primary antibody against Flag-tag, sections were incubated with the fluorescent secondary antibody Alexa Fluor Plus 594 diluted at 1:1500 (A32740, Invitrogen, Carlsbad, CA, USA). The cytoskeleton was then stained with a FITC-conjugated anti-beta actin mouse monoclonal antibody diluted at 1:200–400 (ab6277, Abcam, Cambridge, MA, USA), followed by nuclear staining by DAPI (P-36931, Invitrogen). Imaging was conducted using a Leica STELLARIS 8 confocal microscope with HyD detectors (Leica, Teaneck, NJ, USA) at the Imaging Laboratory of the University of Michigan Diabetes Research Center.

### 4.6. Citrate Assay

The developing enamel matrix on the mandibular first permanent molars of 6-month-old pigs was dissected free of soft tissues and surface-cleaned with Kimwipes. Both soft and hard enamel matrices were separately scraped from the surface, collected, and weighed, and 6 soft enamel (20.1~23.5 mg) and 6 hard enamel (21.7~24.6 mg) samples were suspended in 200 µL of PBS. After homogenization and centrifugation, the supernatant (containing free citrate) was collected. The insoluble pellet (containing bound citrate) was dissolved with 400 µL of 1 N HCl for 5 h at 4 °C and centrifuged, and 4 µL of supernatant was neutralized with 196 µL of 30 mM Tris-HCl buffer (pH 8.8). Both free and bound citrate assays were carried out by using 20 µL of each sample. The citrate concentration was determined using the EnzyChrom Citrate Assay Kit (BioAssay Systems ECIT100, Fisher Scientific, Waltham, MA, USA). A standard curve was first generated with a linear detection range of 0 to 60 μM. The colorimetric intensity at an absorbance of 570 nm for each sample was recorded, and the citrate concentration of each sample was normalized by its weight and calculated by direct comparison to the concentrations plotted in the standard curve.

To compare these results (expressed in µmol/g) to units in mM (used for non-mineralized tissues), we assumed a sample density of 1 g/mL or 1000 g/L (this assumption is only an estimate as the densities of secretory- and maturation-stage enamel are not identical). Thus, 5.019 µmol/g = 5.019 × 10^−6^ mol/g × 1000 g/L = 5.019 × 10^−3^ mol/L = 5.019 mM. To determine the percentage of citrate in enamel, we used the molecular weight of citrate (192 g/mol) to compute 5 µmol/g × 192 g/mol × 1/1,000,000 µmol = 0.00096 g (citrate)/g (enamel) = 0.096% citrate by weight. One-way ANOVA was conducted to compare the citrate contents in the soft and hard enamel.

## Data Availability

The mouse strain, MMRRC_068041-UNC (B6.Cg-*Slc13a5*^tm1Jcch^/Mmnc), is available at the Mutant Mouse Resource and Research Center—UNC facility. Study data of *Slc13a5*^+/+^, *Slc13a5*^+/Flag^, and *Slc13a5*^Flag/Flag^ are available at FaceBase, data set 1-Y7T8 entitled “Generation and Characterization of a Slc13a5 Knock-in Mouse Model with C-terminus Flag”.

## Supplementary Materials

The following supporting information can be downloaded at https://www.mdpi.com/article/10.3390/ijms26146707/s1.

## Abbreviations

The following abbreviations are used in this manuscript:

bSEM	Backscattered scanning electron microscopy
DEE25	Developmental and epileptic encephalopathy 25 with amelogenesis imperfecta
IEDs	Inherited enamel defects
MMRRC	Mutant Mouse Resource and Research Center
