# Supplementary material for: Citrate Transporter Expression and Localization: The Slc13a5Flag Mouse Model
Source: Int J Mol Sci. 2025 Jul 12;26(14):6707. doi: 10.3390/ijms26146707 (PMC12294767; doi:10.3390/ijms26146707)
Supplement: Supplementary file 1 [file ijms-26-06707-s001.zip › ijms-3682833-supplementary.pdf]

# Citrate Transporter Expression and Localization: The *Slc13a5*$^{Flag}$ Mouse Model

Jan C-C. Hu[1*], Tian Liang[2], Hong Zhang[1], Yuanyuan Hu[2], Yasuo Yamakoshi[3], Ryuji Yamamoto[3], Chuhua Zhang[1], Hui Li[1], Charles E. Smith[1,4], and James P. Simmer[1]

1. Department of Biologic and Materials Sciences & Prosthodontics, University of Michigan School of Dentistry, 1011 N University Ave. Ann Arbor, MI 48109, USA.
2. Department of Orthodontic and Pediatric Dentistry, University of Michigan School of Dentistry, 1011 N University Ave. Ann Arbor, MI 48109, USA.
3. Department of Biochemistry and Molecular Biology, School of Dental Medicine, Tsurumi University, 2-1-3 Tsurumi, Tsurumi-ku, Yokohama 230-8501, Japan.
4. Department of Anatomy & Cell Biology, Faculty of Medicine & Health Sciences, McGill University, 3640 University St., Montreal, QC H3A 0C7, Canada.

**Email addresses of authors:**
janhu@umich.edu; tianl@umich.edu; zhanghon@umich.edu; yyhu@umich.edu; yamakoshi-y@tsurumi-u.ac.jp; yamamoto-rj@tsurumi-u.ac.jp; chuhuaz@umich.edu; lihuium@umich.edu; charles.smith@mcgill.ca; jsimmer@umich.edu

**Supplementary Figure S1.** Homology arm recombination introduced sequence changes to *Slc13a5* intron 11 and exon 12**.**

**Supplementary Figure S2:** *Slc13a5* gene expression pattern and intensity in D4 maxillary molars.

**Supplementary Figure S3:** *Slc13a5*$^{Flag}$ gene expression assessed by RT-PCR

**Supplementary Figure S4:** Expression of *Slc13a5* in testis of two-month-old wild-type mice.

A

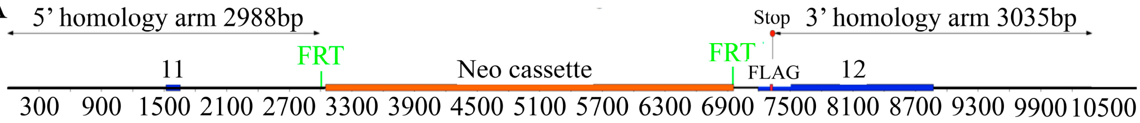


B

*Slc13a5*$^{+/+}$ NCBI Reference Sequence: NC_000077.7 nucleotides (23296-23946) (651 bp)

ATGCTGCAGATTTGATTCTGGACCACAGGAAAAATTCCATGCTGGGGTGTTGGAAAAATGTCTCATGAGTGTCAGGAAGGCA
TACAAAATGGGTGGCAGAAAGAGCTATGGGAGAGTCACGAGACGTGGCTGATGTCTTACACCAGCTCTCTGAAGTCACTTTC
CAACATCACAATGGGTGTAGAGAACCCACCGTGCTGGCTCCTTCCAAGGGATTCTATCAGCGACAATGACACTTAATTTGCT
TTGTCCTGAGACGAGAGGAGCCCAGTGTGCAGGAACACAAGGCCCAGTCAAGACCTGAGTTGCTGACTGATCCTGTTTTTAC
TCCCTAGATGAAAACAGGATTGATAATGAACTTCGTTGGAATCCTATCTGTGTTTCTGTCAGTCAACACCTGGGGTCGGGCT
ATGTTTAACTTGGATAACTTCCCCGACTGGGCAAATTCAACAAGTGTTAACACTTAGGAAGAGCCGCAAGAGCACAAGCATG
CCCCCCCCACCCCAACCCTTTTGAGGACTATGAACCTTCTGGCACACCTTGCACAGAGCACTGGTGCTCACACTCTAGTGTG
ATCCAATGATGTCAACACCCCAAGAAGATCTACCCAACTATATCAAGTTCAGAGATGGCAATGGATGATGGGAAGAT

*Slc13a5*$^{Flag/Flag}$ (773 bp)

ATGCTGCAGATTTGATTCTGGACCACAGGAAAAATTCCATGCTGGGGTGTTGGAAAAATGTCTCATGAGTGTCAGGAAGGCA
TACAAAATGGGTGGCAGAAAGAGCTATGGGAGAGTCACGAGACGTGGCTGATGTCTTACACCAGCTCTCTGAAGTCACTTTC
CAACATCACAATGGGATAGGCGCGCCGAAGTTCCTATTCCGAAGTTCCTATTCTCTAGTAAGTATAGGAACTTCTTATGATC
TAAGGTACCTGTAGAGAACCCACCGTGCTGGCTCCTTCCAAGGGATTCTATCAGCGACAATGACACTTAATTTGCTTTGTCC
TGAGACGAGAGGAGCCCAGTGTGCAGGAACACAAGGCCCAGTCAAGACCTGAGTTGCTGACTGATCCTGTTTTTACTCCCTA
GATGAAAACAGGATTGATAATGAACTTCGTTGGAATCCTATCTGTGTTTCTGTCAGTCAACACCTGGGGTCGGGCTATGTTT
AACTTGGATAACTTCCCCGACTGGGCAAATTCAACAAGTGTTAACACTGACTACAAAGACGATGACGACAAG**TAG**GCCAAGC
AGGCTTAATTAACCGAAAGAGCCGCAAGAGCACAAGCATGCCCCCCCCACCCCAACCCTTTTGAGGACTATGAACCTTCTGG
CACACCTTGCACAGAGCACTGGTGCTCACACTCTAGTGTGATCCAATGATGTCAACACCCCAAGAAGATCTACCCAACTATA
TCAAGTTCAGAGATGGCAATGGATGATGGGAAGAT

**Supplementary Figure S1. Homology arm recombination introduced sequence changes to *Slc13a5* intron 11 and exon 12.** (A) Segment of the mouse *Slc13a5* gene spanning from the 3' end of intron 10 to beyond the coding region of exon 12 (the most 3' exon). The Neo cassette (orange) is shown flanked by FRT sites. The FLAG coding sequence is added to the 5' end of exon 12 (blue) upstream of the translation termination codon. (B) *Top:* Wild-type *Slc13a5* gene sequence showing the 3'end of intron 11 and exon 12 (cyan). The GT highlighted in yellow is where the FRT-Neo cassette-FRT sequence was inserted. Underlined sequences are PCR primer annealing sites. The TTAGGA sequence is the natural stop codon (underlined) where the Flag coding sequence was inserted. *Bottom:* The first sequence in magenta is what remains in intron 11 after the Neo cassette was removed by FRT recombination. The second magenta sequence in exon 12 contains the T followed by the Flag coding sequence ending with a **TAG** stop codon and followed by 23 additional nucleotides. The two sequences in magenta include the entire differences between the Slc13a5 wild-type gene and Slc13a5$^{Flag}$. Size of amplification product including the two underlined sequences: *Slc13a5*$^{+/+}$: 365 bp; *Slc13a5*$^{Flag/Flag}$: 411 bp were used to distinguish modified from wild-type sequences.

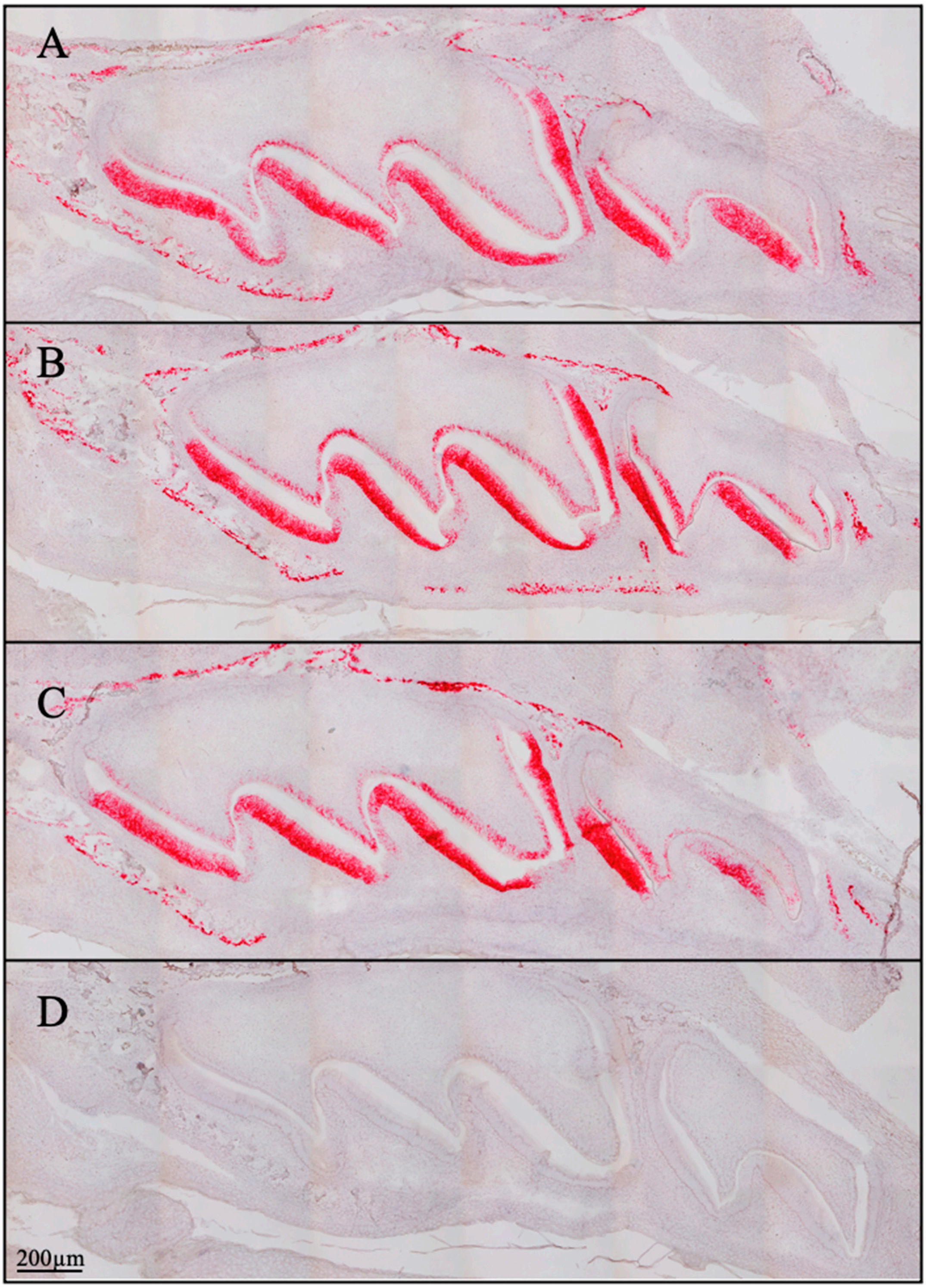


**Supplementary Figure S2. Comparable *Slc13a5 in situ* hybridization patterns in D4 maxillary molars** from (A) wild-type, (B) *Slc13a5*$^{+/Flag}$, and (C) *Slc13a5*$^{Flag/Flag}$. (D) negative control with no primary antibody. The pattern and intensity of *Slc13a5* transcript expression was comparable among the samples from all three genotypes indicating that addition of the Flag coding sequence did not alter *Slc13a5* mRNA expression or stability.

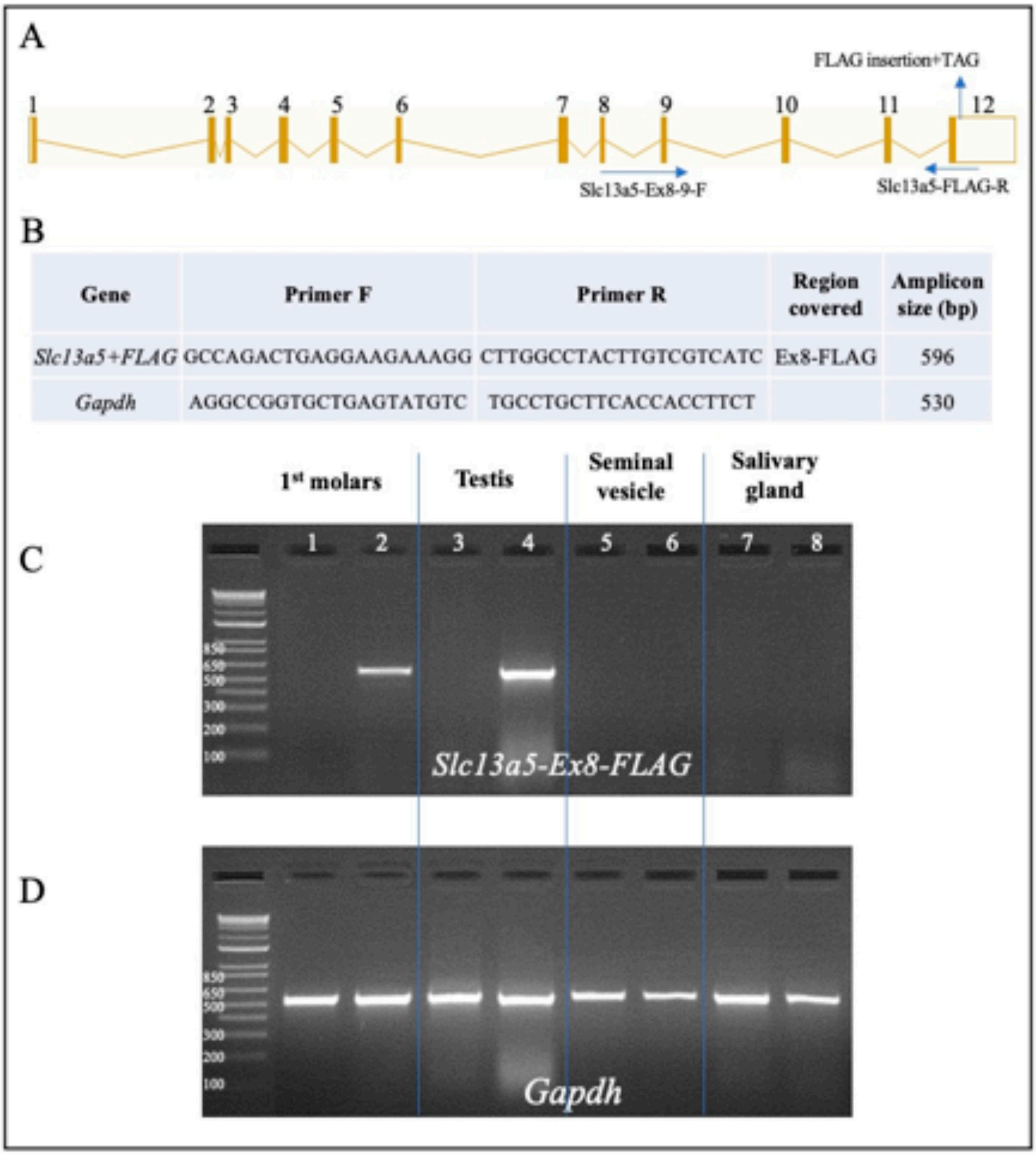


**Supplementary Figure S3.** *Slc13a5*$^{\mathrm{Flag}}$ gene expression was assessed by RT-PCR of molars from D5 from wild-type (Slc13a5$^{+/+}$) and newborn *Slc13a5*$^{\mathrm{Flag/Flag}}$ mice. Salivary gland, testis, and seminal vesicle were from 7-week-old *Slc13a5*$^{+/+}$ mice and 32-week-old *Slc13a5*$^{\mathrm{Flag/Flag}}$ mice. (A) The mouse *Slc13a5* gene structure from ENSMUST00000021161.14 was used to depict the PCR primer locations. (B) PCR primer sequences and amplicon sizes (C) The *Slc13a5*$^{\mathrm{Flag}}$ gene was expressed in the developing first molars and adult testis but not in the adult salivary gland or seminal vesicle. (D) *Gapdh* was amplified to demonstrate RNA quality and quantity control. Odd number lanes were the wild-type samples and even number lanes were the *Slc13a5*$^{\mathrm{Flag/Flag}}$ samples.

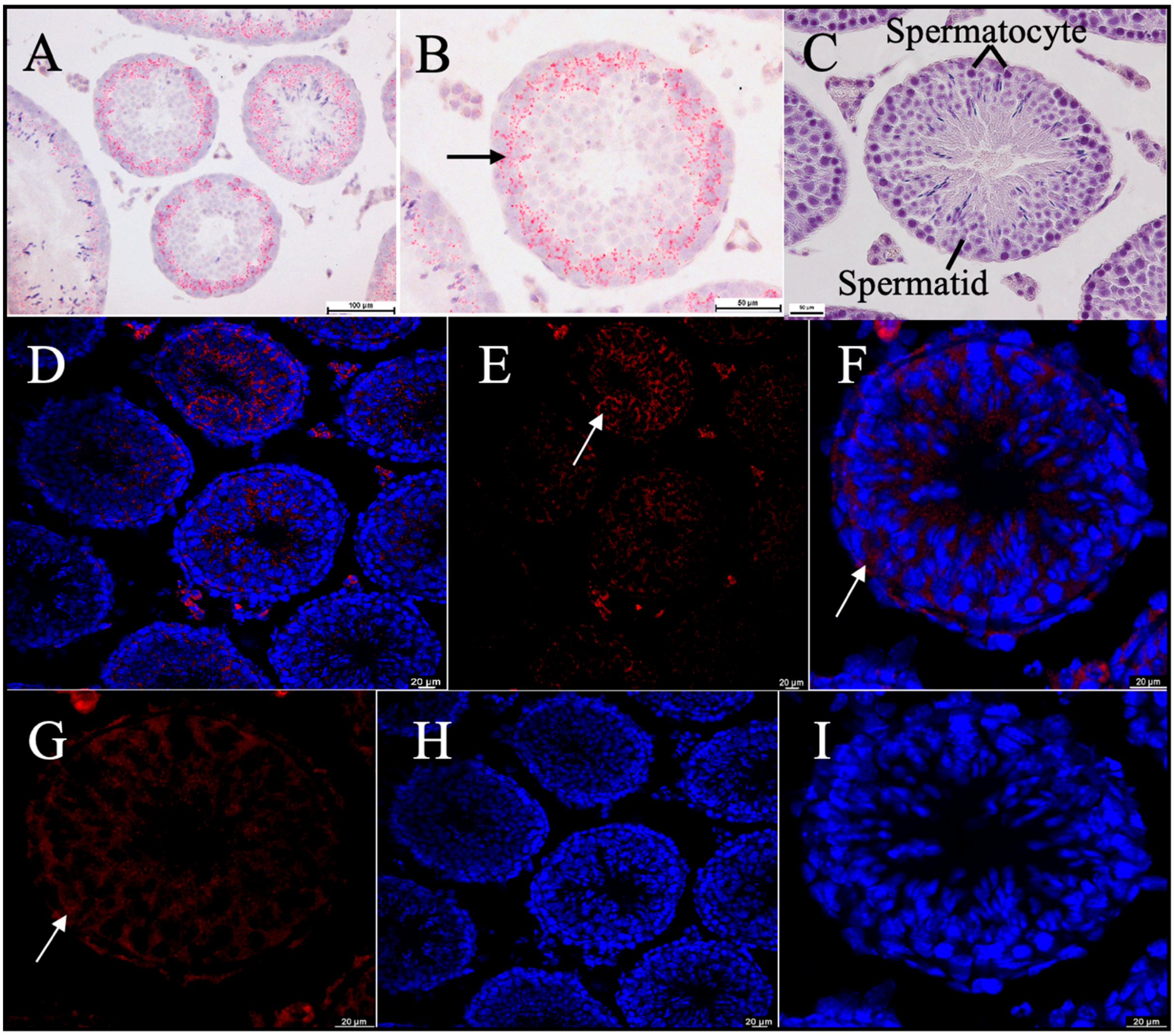


**Supplementary Figure S4. Expression of *Slc13a5* in testis of two-month-old wild-type mice.** Positive expression was detected in the spermatocytes using RNAScope *in situ* hybridization (A-B, arrow). No signal was detected in a negative control without the *Slc13a5* probe (C). (D-G) Determining the protein expression of *Slc13a5*$^{\text{Flag}}$ by immunofluorescent detection using anti-Flag PA1-984B antibodies, we observed positive staining in spermatocytes (D-G, arrows) which validated the *in situ* and RT-PCR findings. Combined Flag (red) and DAPI (blue) signal in D&E (low magnification) and F&G (high magnification); Flag signal detected only in D-G; Negative Flag signal in experimental control without Flag antibody, H (low magnification) & I (high magnification).
